# Chinese medical students’ agreement with and fulfillment of the *Physician Charter*

**DOI:** 10.1186/s12909-018-1324-x

**Published:** 2018-09-17

**Authors:** Ning Ding, Dan Yan, Honghe Li, Yuan Ma, Deliang Wen

**Affiliations:** 1 Institute for International Health Professions Education and Research, China Medical University, No.77 Puhe Road, Shenyang North New Area, Shenyang, Liaoning Province People’s Republic of China; 2 School of Public Health, Dalian Medical University, Dalian, Liaoning China; 3 Office of Student Affairs, China Medical University, Shenyang, Liaoning China

**Keywords:** Medical professionalism, *Medical professionalism in the new millennium: A physician charter*, The *Physician Charter*, Medical professionalism education, China

## Abstract

**Background:**

Although it has been nearly 15 years since the *Medical Professionalism in the New Millennium: A Physician Charter* (the *Physician Charter*) was proposed to reaffirm medical professionalism in response to the new challenges in healthcare delivery in the new century, the manner in which Chinese medical students agree with and fulfill the principles and responsibilities of professionalism defined in the *Physician Charter* still remains unknown.

**Methods:**

In March 2016, 748 fifth-year medical students from China Medical University (CMU) participated in a survey in which they indicated their rate of agreement with and manner of fulfillment of the principles and responsibilities defined in the *Physician Charter* using a 10-point Likert scale. The data were then analyzed by t-tests, exploratory factor analysis, and multiple linear regressions.

**Results:**

The total score of agreement with the *Physician Charter* was significantly higher than that of fulfillment (*p* < 0.001). The largest difference between agreement and fulfillment scores were with the principle of social justice (P3), commitments to improving access to care (R6), and a just distribution of finite resources (R7). Exploratory factor analysis distinguished two principles - primacy of patient welfare (P1) and patient autonomy (P2) - from the others in terms of the gap between agreement and fulfillment. This is partially because the proportion of students who rated agreement lower than fulfillment of P1 or P2 was much higher than it was for any other principle or responsibility. Additionally, multiple linear regressions show that students who are enrolled in a five-year program or who was registered as a rural resident (i.e. holding a rural *Hukou)* had significantly higher scores of agreement, but not fulfillment.

**Conclusions:**

Chinese medical students endorsed the *Physician Charter* and its core values of medical professionalism, although they felt difficult to fulfill in practice. Medical educators and the health authority should act together to support and foster professional values.

**Electronic supplementary material:**

The online version of this article (10.1186/s12909-018-1324-x) contains supplementary material, which is available to authorized users.

## Background

Faced with challenges in healthcare delivery in the era of a technology explosion and rapid changes in the market structure of the healthcare industry, the American Board of Internal Medicine (ABIM), the American College of Physicians-American Society of Internal Medicine Foundation (ACP-ASIM Foundation), and the European Federation of Internal Medicine (EFIM) jointly proposed *Medical Professionalism in the New Millennium: A Physician Charter* (the *Physician Charter*, hereafter) to reaffirm the fundamental and universal principles and values of medical professionalism [1, 2]. Once after released, the *Physician Charter*, comprising three fundamental principles and ten professional responsibilities, has been endorsed by more than 130 organizations and millions of copies in various languages have been distributed worldwide [3].

As the only Chinese organization that has endorsed the *Physician Charter*, in 2006 the Chinese Medical Doctor Association (CMDA) had the charter translated to Chinese [4], prompting efforts of researchers to investigate various aspects of medical professionalism in the context of China. For example, Chen et al. studied students’ thoughts on the relationship between physicians and patients in China [5]. Additionally, Li et al. evaluated and compared the professionalism status of medical students enrolled into different programs [6, 7] and Wen et al. investigated one component of professionalism, specifically empathy, in Chinese physicians and medical students [8, 9].

Furthermore, while some Chinese medical schools/universities embraced medical professionalism and fostered professionalism in their students to enhance their competency in their future careers [10], to the best of our knowledge, only a few Chinese medical schools adopted the *Physician Charter*’s principles and responsibilities into their curriculums and few, if any, provided a systematic education of professionalism. For example, China Medical University (CMU) was the first medical university established by Communist Party of China. In order to engrain the idea of professionalism in medical students, CMU added the *Physician Charter* into its orientation course of medicine. This, however, is the only course explicitly related to professionalism in the curriculum of medical students.

Role modeling and personal reflections, guided by faculty, on the cultivation of professionalism during clerkship and residency are effective techniques for developing professionalism [11]. However, many Chinese clinical supervisors and residency trainers hold the opinion that clinical training’s main aim is to enrich leaners’ personal practice capacities, skills, and experiences and the enhancement of professionalism is only a byproduct [12]. As a result, during this stage, professionalism in China is normally self-learned by the students and the efficiency and effectiveness of this process are significantly impaired.

Although it has been more than ten years since CMDA endorsed the *Physician Charter*, the education of medical professionalism in China is still not systematically engrained and is therefore of limited availability. Considering that professionalism is a core competency of medical students and is closely related to the wellbeing of patients, we investigated the agreement with and fulfillment of the principles and responsibilities defined in the *Physician Charter* in medical students from China Medical University. We anticipated that this study may aid institutions with identifying any necessary reforms in the education frameworks of medical professionalism in China and may also be of value to countries aiming to improve their own medical professionalism.

## Methods

### Study design and sample

All 880 fifth-year medical students of 2015/16 enrolled in either the five-year or seven-year medical education program of China Medical University (CMU) were invited to participate in this study in March of 2016, which was the beginning of the second semester of the academic year. The beginning of the second semester of year five is an ideal time to assess agreement with and fulfillment of the *Physician Charter,* because all students have completed their preclinical study and have had some clerkship training. In addition, this timing also provides us with an opportunity to quantify the effect of clerkship on professionalism because at that time, the fifth-year students have already finished one-semester of clinical clerkship, whereas the seventh-year students have just begun their clerkship. Subsequently, there was a nearly 20-week difference in the clinical experience of the medical students at the time of survey, which may influence their levels of the agreement or fulfillment of the *Physician Charter*.

The study was approved by the Institutional Review Board of China Medical University. All participants signed an informed consent form, in which confidentiality was guaranteed.

### Survey instruments

#### Medical professionalism in the new millennium

*A Physician Charter (the Physician Charter)* contains 13 items, including three fundamental principles and ten professional responsibilities (see Table 1) [1, 2]. The *Physician Charter* was translated into Chinese in 2006 by the Chinese Medical Doctor Association (CMDA) and was used in the present study. All participants were asked to evaluate their agreement and fulfillment of each principle and responsibility using a 10-point Likert scale. Specifically, participants quantified the extent to which they agreed or disagreed with each principle or responsibility on a scale ranging from one (strongly disagree) to ten (strongly agree). Participants were then asked the extent to which they fulfilled each item in clinical practice on a scale ranging from one (cannot fulfill at all) and ten (can perfectly fulfill).

**Table 1:** Medical Professionalism in the New Millennium: A Physician Charter

Item	Content
Fundamental Principles	
P1	Principle of primacy of patient welfare.
P2	Principle of patient autonomy.
P3	Principle of social justice.
Professional Responsibilities	
R1	Commitment to professional competence.
R2	Commitment to honesty with patients.
R3	Commitment to patient confidentiality.
R4	Commitment to maintaining appropriate relations with patients.
R5	Commitment to improving quality of care.
R6	Commitment to improving access to care.
R7	Commitment to a just distribution of finite resources.
R8	Commitment to scientific knowledge.
R9	Commitment to maintaining trust by managing conflicts of interest.
R10	Commitment to professional responsibilities.

Participants were also asked to indicate their gender, age, the program enrolled (five- or seven-year), and whether they hold a rural *Hukou*. In China, *Hukou* is a record in the household registration system that officially identifies a person as a resident of an area. Because Chinese urban residents receive benefits ranging from education, health care, to retirement pension and rural citizens do not frequently receive these benefits, the *Hukou* status is often used as a benchmark as to the degree of social welfare received.

Furthermore, all participants provided consent to allow access to the participant’s status as a student leader, the receipt of government poverty subsidies, and their overall academic performance rank from the student administration office of CMU.

### Statistical analysis

First, the Kaiser-Meyer-Olkin (KMO) measure and the Bartlett’s test for sphericity were used to demonstrate the appropriateness of the utilization of factor analysis. We then used Cronbach’s α to test the reliability of the question set that originated from the *Physician Charter.* Exploratory factor analysis, specifically principal component analysis (PCA), was used to investigate the internal structure of Chinese medical students’ agreement on and fulfillment of the *Physician Charter* and any gaps between these two ratings. When more than one factor was identified, a direct oblimin rotation was used to account for the possible correlation between factors [13]. Although we used this factor analysis technique to simplify the data and manifest its structure, there was never an intent to develop any instrument to assess students’ professionalism behavior or attitude, so the reliability of the question set originating from the *Physician Charter* is not the focus of the present study.

The overall scores of agreement with and of fulfillment of the *Physician Charter* were calculated by summing all corresponding individual scores. The gaps between agreement and fulfillment were assessed by t-tests.

Finally, the associations between demographic and economic characteristics and the overall scores of agreement and fulfillment were modeled with multiple linear regressions [14]. The covariates included being female, age (in years) and its quadratic term, whether enrolled in the five-year program, whether holding a rural *Hukou*, excellent academic performance (a dummy variable was created to identify if the students’ academic score was in the top quarter or not), identification as a student leader, and whether they are from a household who has received government poverty subsidies. Missing values were imputed with median scores. A robustness check was done by excluding all samples with missing values to evaluate potential bias caused by missing data.

All was analyses were implemented with Stata 13.0 [15].

## Results

Most (*N* = 806) fifth-year medical students returned their questionnaires, with 85% (*N* = 748) of all fifth year student of CMU completing the question set originating from the *Physician Charter*.

There were very few missing values on variables, including gender (0.5%), age (1.2%), education program (1.6%) and *Hukou* status (4.4%). The summary statistics in Table 2 show that among the 748 medical students, nearly 60% were female, more than half were enrolled in the five-year program, and more than one quarter were from poor families or rural areas.

**Table 2:** Descriptive characteristics

Variable	N	Percent (%)
Gender		
Male	302	40.37
Female	446	59.63
Medical education Program		
Five-year	401	53.61
Seven-year	347	46.39
Student leaders		
No	506	67.65
Yes	242	32.35
Receipt of government poverty subsidies		
No	552	73.8
Yes	196	26.2
*Hukou*		
Urban	536	71.66
Rural	212	28.34

All of the KMO measures were larger than 0.9 and all results of Bartlett’s tests were significant, suggesting that the data used in the present study were appropriate for factor analysis (see Additional file 1). Additionally, all values of Cronbach’s α were greater than 0.9, suggesting the question set based on the *Physician Charter* is reliable (see Additional file 1).

The results of exploratory factor analysis for the agreement of the *Physician Charter* are shown in Table 3. Although only one factor, with an eigenvalue larger than one, was identified, nearly 70% variance can be explained with that factor. All factor loadings were very high (≥0.75) and close to each other, with the exception of P1 and P2 (principle of primacy of patient welfare and principle of patient autonomy), which had factor loadings of 0.62 and 0.69, respectively. The results of fulfillment, shown in Table 3, are very similar in that only one factor can be identified, more than 60% variance is explained, factor loadings were all high, and again, P1 and P2 demonstrated the lowest factor loadings.

**Table 3:** Structures of the agreement with and fulfillment of the *Physician Charter* among 748 Chinese medical students from CMU in China, exploratory factor analysis, factor matrix

Item	Agreement	Fulfillment
P1	0.62	0.60
P2	0.69	0.71
P3	0.87	0.77
R1	0.91	0.82
R2	0.88	0.84
R3	0.89	0.81
R4	0.85	0.82
R5	0.92	0.86
R6	0.80	0.74
R7	0.79	0.75
R8	0.90	0.83
R9	0.77	0.78
R10	0.91	0.81
Eigenvalue	9.04	7.98
% Variance explained	69.53	61.42

**Fig. 1:**
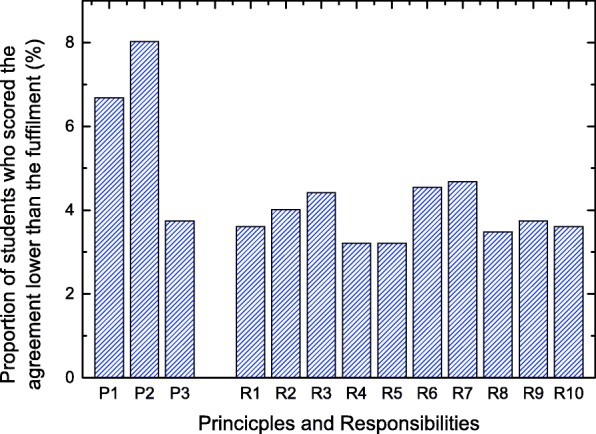
Proportion of students who rated their agreement lower than their fulfillment in terms of each principle or responsibility defined in the *Physician Charter*

Two factors were identified from the exploratory factor analysis on the gaps between agreement and fulfillment (Table 4). Factor 1 was driven by P3, the principle of social justice, with all of the responsibilities accounting for 48.77% of the total variance. Factor 2, driven mainly by P1 and P2, accounted for 9.34% of the variance. To explore the potential cause of this division, the proportion of students who scored the agreement of each principle lower than either the fulfillment of each principle or responsibility of the *Physician Charter* was presented in Fig. 1. Clearly, for P1 and P2, which are drivers of Factor 2, the number of such inversions is significantly larger.

**Table 4:** Structure of the difference between agreement with and fulfillment of the *Physician Charter* among 748 Chinese medical students, exploratory factor analysis, rotated factor matrix

Item	Factor	
	1	2
P1		0.57
P2		0.63
P3	0.64	
R1	0.70	
R2	0.72	
R3	0.69	
R4	0.73	
R5	0.83	
R6	0.76	
R7	0.76	
R8	0.81	
R9	0.77	
R10	0.70	
Eigenvalue	6.34	1.21
% Variance explained	48.77	9.34

Values less than 0.55 are not shown

The summary of agreement and fulfillment scores in Table 5 shows that the overall score of agreement was as high as 111.07 out of total score 130, suggesting that medical students from CMU acknowledge the *Physician Charter*. The average score of agreement for each principle or responsibility varied from 7.57 (P1, principle of primacy of patient welfare) to 9.00 (R10, commitment to professional responsibilities.). The scores of fulfillment were much lower than the scores of agreement, but were still reasonable, with an overall score of 88.22. The lowest score was given to P1, whereas the highest scores were R10 and R3 (commitment to patient confidentiality). Table 5 shows the average difference between agreement and fulfillment. All differences were significantly larger than zero (ps < 0.001), therefore t- values were omitted. All differences between agreement and fulfillment were smaller than 2, except for R7 (commitment to a just distribution of finite resources), R6 (commitment to improving access to care), and P3 (principle of social justice).

**Table 5:** Agreement with and fulfillment of the *Physician Charter* and their difference among 748 Chinese medical students

Item	Agreement	Fulfillment	Gap
	Mean(SD)	Mean(SD)	Mean(SD)
Overall	111.07 (20.23)	88.22 (21.31)	22.84 (16.83)
Fundamental Principles			
P1	7.57 (2.25)	5.71 (2.08)	1.86 (2.13)
P2	8.11 (2.03)	6.63 (2.08)	1.47 (2.08)
P3	8.76 (1.78)	6.66 (2.04)	2.10 (1.89)
Professional Responsibilities			
R1	8.86 (1.81)	7.00(2.04)	1.86 (1.91)
R2	8.54 (1.81)	6.86 (2.06)	1.68 (1.87)
R3	8.88 (1.72)	7.60 (2.14)	1.27 (1.61)
R4	8.45 (1.90)	7.05 (2.06)	1.39 (1.60)
R5	8.84 (1.72)	7.17 (1.97)	1.67 (1.58)
R6	8.39 (2.09)	6.27 (2.22)	2.11 (2.00)
R7	8.43 (2.07)	6.17 (2.26)	2.26 (2.20)
R8	8.80 (1.76)	7.02 (2.07)	1.77 (1.76)
R9	8.47 (2.01)	6.50 (2.25)	1.96 (1.92)
R10	9.00 (1.64)	7.58 (2.04)	1.41 (1.56)

Agreement and fulfillment of each principle or responsibility defined in the *Physician Charter* was scored with 10-point Likert scales. All differences between agreement and fulfillment are statistically significant at *p* < 0.001

The results of the multiple linear regressions (see Table 6) demonstrate that the students enrolled in the five-year program had significant higher overall scores of agreement (B = 5.13, Beta = 0.13, *p* = 0.001) than those enrolled in the seven-year program and the scores of those holding rural *Hukou* were significantly higher (B = 4.98, Beta = 0.11, *p* < 0.01) than those not. However, none of the personal characteristics significantly affected the overall score of fulfillment. The results of the robustness check based on the sample with complete information retain the same pattern (see Additional file 2).

**Table 6:** Effects of demographic and economic characteristics on overall scores of agreement with and fulfillment of the *Physician Charter* among 748 Chinese medical students, multiple linear regressions

Variable	Agreement					Fulfillment		
	B	SE	Beta	*p*-value	B	SE	Beta	*p*-value
Female	0.77	1.55	0.02	0.62	−0.24	1.65	−0.01	0.88
Age (year)	15.27	16.05	0.71	0.34	6.44	17.07	0.28	0.71
Age^2^	−0.32	0.34	−0.68	0.36	−0.13	0.37	−0.27	0.72
Five-year program	5.13	1.49	0.13	0.001	3.03	1.58	0.07	0.06
Rural *Hukou*	4.98	1.80	0.11	0.006	2.93	1.92	0.06	0.13
Excellent academic performance	−2.13	1.80	−0.05	0.24	−1.99	1.91	− 0.04	0.30
Student leader	−0.26	1.65	−0.01	0.88	−1.25	1.75	−0.03	0.48
Receipt of government poverty subsidies	−2.85	1.87	−0.06	0.13	−3.00	1.99	−0.06	0.13
Constant	−75.32	187.49		0.69	9.87	199.29		0.96

## Discussion

In this study, we investigated CMU’s fifth-year medical students’ agreement and fulfillment of the Physician Charter. The results show that Chinese medical students respected and embraced the *Physician Charter* and its core value of medical professionalism. However, their results suggest that they may feel that it is difficult to fulfill the principles and responsibilities as defined in the *Physician Charter* in practice.

The findings that students respected professionalism are not surprising because first, despite the differences in values and norms between China and the western world, views on medical professionalism are quite similar. These views can be traced back to Simiao Sun, a famous doctor in Tang Dynasty, who stated that a good doctor should possess not only distinguished medical knowledge and skills, but should also have a noble character [16]. He also believed that the interests of the patient should be the priority of doctors, rather than the economic or social returns from patient care. Indeed, he believed that doctors should sacrifice their own interests for that of the patients, if necessary. Given this background, it is easy for medical students in China to accept the *Physician Charter,* having been instilled with these ideas for many years. Another reason that medical students in China so readily accept the *Physician Charter* can be attributed to the efforts to disseminate the *Physician Charter* once the Chinese Medical Doctor Association (CMDA) endorsed it in 2005, allowing medical students in China have plenty of opportunities to become familiar with the *Physician Charter*.

Difficulties in fulfilling the principles and responsibilities defined in the *Physician Charter* are well documented. For instance, a United States survey in 2007 demonstrated that physicians agreed with some of the *Physician Charter*’s principles, but their self-reported behavior often violated those values [17]. Combined with findings of this study, it might be concluded that the gap between the ideals espoused in the *Physician Charter* and the behavior in practice might exist at the very beginning of their career. Therefore, an integrity value system should be established before physicians start practicing.

Chinese medical students indicated that the largest gaps between agreement and fulfillment lied in P3 (principle of social justice), R6 (commitment to improving access to care), and R7 (commitment to a just distribution of finite resources), underlining two main challenges, efficiency and equity, faced by the Chinese government and many health authorities, globally. The former, characterized by R6, is defined by the drive to have an optimal allocation of finite resources to maximize production, healthcare in this case. The latter, closely related to P3 and R7, is related to the issues surrounding how to distribute healthcare throughout the whole society. It is obvious that, except for the individual efforts of physicians and other healthcare workers, the government should ensure sufficient investments in healthcare, stimulate progress in health technologies, and, more importantly, implement institutional reforms to ensure equity in health care delivery when necessary.

Another valuable finding, which might shed light on medical professionalism education in China, is that exploratory factor analysis distinguished the principle of primacy of patient welfare (P1) and patient autonomy (P2) from the other principle or any responsibility of the *Physician Charter* in terms of the gap between the students’ agreement and their fulfillment. Detailed analysis shows that the proportion of those whose agreement score was lower than their fulfillment scores for P1 or P2 was significantly higher than those of any other principle or responsibility. This suggests that a small group of medical students, did not embrace P1 or P2, but thought they could adhere to the principles if they were willing. However, it is also possible that students just did not agree with the manner in which these principles were expressed in the charter, but agreed with the actual principles in the *Physician Charter*. For example, it is reasonable to argue that if the cost of adherence to P2 is the sacrifice of patients’ lives, respect for patient autonomy is not so important and is indispensable in ethical practice. However, students might agree with P2 if the brief expression of P2 is expanded to add the following: the respect is based on actively exchanging ideas, explicitly negotiating differences, and sharing power between patients and physicians [18]; or perhaps after they are informed by both the medical facts and the physician’s experience, patients autonomously make choices. This phenomenon provides evidence of the weakness, if not the absence, of the medical professionalism education in China, which could hamper the development of healthcare. Therefore, universities in China should provide courses on professionalism and teachers should convey the ideas and concepts clearly during such courses.

Finally, as mentioned previously, there is a nearly 20-week difference in the clinical experience between the five- and seven-year program students, which might be the reason why the former had a significant higher overall score of agreement of the *Physician Charter* (by 0.13 SD). Admittedly, the students enrolled in different programs are not perfectly comparable, however, with a near identical curriculum, this comparison can provide us with some valuable insights. Consistent with previous studies, the significant difference in agreement of Physician Charter confirms the importance of clinical clerkship in the professionalism education [19, 20]. It is efficient for medical students to cultivate their professionalism by seeing their senior colleagues’ practicing, reflecting on the models portrayed, and shaping their own behavior to mirror these models [21]. Thus, clinical supervisors in hospitals in China should make the most utility of this hidden curriculum and provide students with sufficient opportunities to learn professionalism in practice.

The conclusions that can be drawn from our study are limited to the extent to which we used cross-sectional data from only one Chinese medical university, examined medical students’ understandings of professionalism from the perspective of the *Physician Charter,* and were not able to symmetrically assess students’ professionalism or to quantify the impacts of curriculums on their behavior after they began practicing medicine. The international and intra-national cultural differences should be taken into account by implementing international comparative analysis and by conducting a nationally representative survey in the future. Another limitation is that nearly 15% of the students did not complete the questions set based on the *Physician Charter*. The main reason for the failure to complete the questionnaires is that many students were doing their internship in hospitals and some students in hospitals far from CMU failed to participate the survey. Although all students of CMU were randomly assigned to their internship hospital and we therefore do not believe the missing data to impact our conclusions given no systematic difference between those assigned to hospitals near CMU and those assigned to hospitals far from CMU, this shortcoming must be addressed in future studies.

## Conclusions

We found that, although the majority of medical students in China embraced the principles espoused in the *Physician Charter*, the gaps between their agreement and fulfillment were still significant. Even nearly 15 years after the publication of the *Physician Charter*, the same challenges remain. The educators of medical professionalism in China, both teachers in the universities and clinical supervisors in hospitals, should make more efforts to precisely convey the core values of professionalism to students. Government should also play a vital role in shaping the healthcare environment to support and foster professional values and to introduce institutional settings to ensure both substantial growth and equity in health care delivery.

## Additional files

Additional file 1: Tests for the measurement properties of the question set originated from the Physician Charter among 748 Chinese medical students. (DOCX 23 kb)

Additional file 2: Effects of demographic and economic characteristics on overall scores of agreement and fulfillment of the Physician Charter among 699 Chinese medical students with complete information, multiple linear regressions. (DOCX 24 kb)

## Abbreviations

ABIM: American Board of Internal Medicine
ACP-ASIM Foundation: American College of Physicians-American Society of Internal Medicine Foundation
CMDA: Chinese Medical Doctor Association
CMU: China Medical University
EFIM: European Federation of Internal Medicine
