# Supplementary material for: Chinese medical students’ agreement with and fulfillment of the Physician Charter
Source: BMC Med Educ. 2018 Sep 17;18:212. doi: 10.1186/s12909-018-1324-x (PMC6142398; doi:10.1186/s12909-018-1324-x)
Supplement: Supplementary file 1 — Tests for the measurement properties of the question set originated from the Physician Charter among 748 Chinese medical students. (DOCX 23 kb) [file 12909_2018_1324_MOESM1_ESM.docx]

### Additional file 1: Tests for the measurement properties of the question set originated from the *Physician Charter* among 748 Chinese medical students

|  | Kaiser-Meyer-Olkin (KMO) measure | Bartlett's test | | Cronbach's $\boldsymbol{\alpha}$ |
| --- | --- | --- | --- | --- |
|  |  | $\boldsymbol{\chi}^{\mathbf{2}}$ | *p-value* |  |
| Agreement | **0.960** | **10202** | **<0.001** | **0.959** |
| Fulfillment | **0.955** | **7304** | **<0.001** | **0.946** |
| Gap | **0.937** | **4862** | **<0.001** | **0.910** |
