# Supplementary material for: Chinese medical students’ agreement with and fulfillment of the Physician Charter
Source: BMC Med Educ. 2018 Sep 17;18:212. doi: 10.1186/s12909-018-1324-x (PMC6142398; doi:10.1186/s12909-018-1324-x)
Supplement: Supplementary file 2 — Effects of demographic and economic characteristics on overall scores of agreement and fulfillment of the Physician Charter among 699 Chinese medical students with complete information, multiple linear regressions. (DOCX 24 kb) [file 12909_2018_1324_MOESM2_ESM.docx]

### Additional file 2: Effects of demographic and economic characteristics on overall scores of agreement with and fulfillment of the *Physician Charter* among 699 Chinese medical students with complete information, multiple linear regressions

| **Variable** | **Agreement** | | |  | | | **Fulfillment** | | |
| --- | --- | --- | --- | --- | --- | --- | --- | --- | --- |
|  | B | SE | Beta | *p-value* |  | B | SE | Beta | *p-value* |
| **Female** | 1.22 | 1.60 | 0.03 | 0.45 |  | 0.51 | 1.69 | 0.01 | 0.77 |
| **Age (year)** | 21.95 | 22.78 | 0.99 | 0.34 |  | -6.78 | 24.08 | -0.29 | 0.78 |
| **Age^2^** | -0.45 | 0.48 | -0.95 | 0.35 |  | 0.15 | 0.51 | 0.31 | 0.76 |
| **Five-year program** | 4.21 | 1.53 | 0.10 | 0.006 |  | 2.07 | 1.62 | 0.05 | 0.20 |
| **Rural *Hukou*** | 5.97 | 1.86 | 0.14 | 0.001 |  | 3.38 | 1.97 | 0.07 | 0.09 |
| **Excellent academic performance** | -1.79 | 1.86 | -0.04 | 0.34 |  | -1.44 | 1.96 | -0.03 | 0.46 |
| **Student cadre** | -0.21 | 1.70 | -0.005 | 0.90 |  | -1.17 | 1.80 | -0.03 | 0.52 |
| **Poor family** | -4.24 | 1.97 | -0.09 | 0.03 |  | -3.22 | 2.08 | -0.07 | 0.12 |
| **Constant** | -159.03 | 268.28 |  | 0.55 |  | 161.72 | 283.59 |  | 0.57 |
